# Scales to Assess Knowledge, Motivation, and Self-Efficacy for HIV PrEP in Colombian MSM: PrEP-COL Study

**DOI:** 10.1155/2021/4789971

**Published:** 2021-09-07

**Authors:** Héctor F. Mueses-Marín, Beatriz Alvarado-Llano, Julián Torres-Isasiga, Pilar Camargo-Plazas, Maria C. Bolívar-Rocha, Ximena Galindo-Orrego, Jorge L. Martínez-Cajas

**Affiliations:** ^1^Corporación de Lucha Contra el Sida, Carrera 56 2-120, Cali, Colombia; ^2^Public Health Science, Queens University, Kingston, Ontario K7L 3N6, Canada; ^3^Montefiore Medical Center, Moses Division, Albert Einstein College of Medicine, The Oval Center at Montefiore, 3230 Bainbridge Avenue, Bronx, NY 10467, USA; ^4^School of Nursing, Queen's University, Kingston, Ontario K7L 3N6, Canada; ^**5**^ Division of Infectious Diseases, Department of Medicine, Queens University, Kingston, Ontario K7L 3N6, Canada

## Abstract

**Objective:**

We evaluated the construct validity Spanish version of knowledge, stigma, norms, and self-efficacy scales regarding PrEP in MSM.

**Methods:**

Sample of 287 MSM. Exploratory confirmatory factor analysis and item response theory were used to validate the constructs. Correlations and confidence interval-based estimation of relevance analyses were conducted to correlate the scales with willingness and intention to use PrEP.

**Results:**

Attitude, stigma, and descriptive and subjective norms scales showed good construct validity and were related to intention and willingness to use PrEP. However, the knowledge scale and self-efficacy scales require further refinement.

**Conclusions:**

The study provides useful information for assessing information, motivation, and self-efficacy related to PrEP use. Our results could be used to test the scales and the theoretical model in other contexts to confirm their usefulness.

## 1. Introduction

As MSM continue to be disproportionately affected by HIV, a combination of preventive strategies, including preexposure prophylaxis (PrEP), is urgently needed to curtail the rate of new HIV diagnoses worldwide. PrEP acceptance in MSM has been low despite years of availability and demonstrated efficacy [1, 2]. In the United States only one of five PrEP-eligible MSM is taking PrEP [3, 4]. Latino MSM in North America had lower PrEP knowledge, acceptance, and uptake [5, 6]. In Latin American countries, where MSM account for 40% of the new cases of HIV each year, PrEP awareness was reported around to 58%, and nearly 52% were willing to use daily PrEP [6]. PrEP uptake is a complex behaviour [1, 7], and the use of a theoretical model to identify appropriate targets for interventions is needed if PrEP is to be adopted widely.

One explanatory model for reducing HIV risk behaviours that has potential in MSM populations is the information motivational behavioural (IMB) model [8]. The IMB model posits that individuals will likely initiate and sustain a behaviour if they are well informed and motivated and perceive themselves as having the skills to initiate and maintain the behaviour. The IMB model has been used to develop HIV prevention interventions proven to be effective in decreasing condomless sex [9] and increasing adherence to antiretroviral therapy [10, 11]. Barriers related to PrEP uptake in MSM are also consistent with the IMB model, including lack of knowledge, negative attitudes, lack of self-efficacy, and low motivation due to stigma [12]. Hence, it is a relevant framework for the development of behavioural interventions for populations at risk of HIV infection and in need of PrEP [12–14].

Identification of PrEP knowledge, motivation, and skills in MSM requires either new instruments or the adaptation and validation of existing ones in different populations. In a recent publication, Walsh [14] used the IMB model to develop new measures for these constructs and relate them to PrEP uptake. The scales assessing knowledge, descriptive norms, and subjective norms have high levels of reliability with alphas of Cronbach greater than or equal to 0.90. The attitude, stigma, and self-efficacy scales had good alphas from 0.79 to 0.87. Confirmatory factor analyses supported the correlation of the scales and the item loading in each a priori latent construct, with most of the scales related to the intention to use PrEP, thus supporting the IMB model. Since the scales have been recently developed and only used in MSM populations living in the United States and are not yet available in Spanish, in this paper, we present the assessment of their internal coherence and construct validity in a sample of MSM from Colombia. In this work, we explored if the items of the scales represent one construct and a relationship exists between the scales and the intention to uptake PrEP. If the PrEP scales are valid and the relations are supported, the results could be used to gain an understanding of PrEP uptake in other Spanish-speaking MSM populations and delineate interventions.

## 2. Materials and Methods

### 2.1. Design

The data from this study came from an online open survey; thus anyone was able to access it by clicking on an online link. The recruitment was between April and October 2020. The link to the online survey was distributed in different ways, including emails to gay and MSM leaders/organizations who were asked to share it with their peers/members through social media, such as Facebook. A total of 7 MSM or transgender-focused organizations posted the study link. The study was also advertised on Grindr for two weeks geographically constricted to three Colombian cities: Cali, Bogota, and Medellin (total population ∼13 million). Individuals who were self-identified as MSM and self-reported an HIV-negative status in the screening questions were prompted to complete the PrEP-related survey. The questionnaire was developed in Qualtrics, was anonymized, and offered no incentives. Participation in the study was voluntary, and access to the questionnaire was granted to those consenting online. The full questionnaire was developed through an iterative process between team members and PrEP experts and was reviewed by community members of the MSM community in three Colombian cities. For this paper, we report selected parts of the questionnaire. This study was reviewed and approved by the Research Ethics Boards of both the Corporación de Lucha contra el Sida (approval certificate no. 034 of May 16, 2018) and Queen's University (DMED-2326-20).

### 2.2. Participants

Participants were eligible for this analysis if they were self-identified as gay/bisexual men, resided in Colombia, and reported to be HIV-negative. The majority of participants reported a negative HIV test in the last 12 months. A total of 287 participants were eligible among 584 who clicked on the questionnaire.

### 2.3. Instruments

Items from the original English scales [14] were translated by one of the team members who is bilingual in Spanish and English and familiar with scale validation (BEA) and back-translated by another team member (JLM) who is also bilingual in Spanish and English and an HIV expert. Then, the translated scales were reviewed using cognitive interview methodology with four MSM, which resulted in some minor changes. These changes included wording, exclusion of two items of the knowledge scale (as they did not apply to the Colombian context), and changing the Likert scale from 5 to 3 answer categories for the scales of attitudes, stigma, and descriptive norms. This latter change sought to decrease the degree of difficulty and time needed for completion. The scales included ten items assessing knowledge on PrEP, five items assessing attitudes, five assessing stigma, six assessing descriptive norms, six assessing subjective norms, and eight items assessing self-efficacy:

 
*Knowledge PrEP Scale*. This scale was initially composed of 13 items but was reduced to 10 items after excluding items related to PrEP coverage, the availability of over-the-counter PrEP, and ways to have access to PrEP in the absence of health insurance. These items did not apply to the Colombian context, as PrEP was not covered nor was it available over the counter in Colombia at the time of the survey. Each item was scored on a 3-point scale: 1 = *true*, 2 = *false*, and 3 = *don*'*t know.* Scores on this scale were recoded as 1 = *correct* and 0 = *incorrect/don*'*t know*, with higher scores indicating a greater degree of knowledge. To reduce the burden of the number of questions, participants were randomly assigned (option available in Qualtrics) to 70% of the items of the knowledge scale, and imputation of nonassigned items was done using the R software (Supplementary Table 1).
 
*PrEP Attitudes.* PrEP attitudes consisted of a 5-item scale, which includes aspects such as perceptions of effectiveness and safety. All original items were translated, and each item was scored on a 3-point scale as 1 = *disagree*, 2 = *neutral*, and 3 = *agree*, with higher scores indicating more positive attitudes.
 
*PrEP Stigma*. This 5-item scale is related to concerns regarding negative perceptions about PrEP use, for instance, the relationship between PrEP use and promiscuity. Each item was scored on a 3-point scale as 1 = *disagree*, 2 = *neutral*, and 3 = *agree*, with higher scores indicating a greater degree of stigma.
 
*Descriptive Norms PrEP*. For this measure, participants were asked to consider the perceptions and interests of friends and people in their community concerning PrEP. Six items were scored on a 3-point scale ranging from 1 = *disagree* to 3 = *agree*, with higher scores indicating more positive norms. This was a change from the 5-point scale used by Walsh [14].
 
*Subjective Norms PrEP*. Participants were also asked to consider the perceptions of friends and their sexual partners. Three items were scored on a 3-point scale ranging from 1 = *disagree* to 3 = *agree*, with higher scores indicating a greater degree of positive norms related to PrEP.
 
*Self-Efficacy PrEP*. This 8-item scale assessed efficacy related to asking about PrEP, adherence to PrEP, and payment of PrEP, among others. Each item was scored on a 4-point scale ranging from 1 = *very difficult* to 4 = *not difficult at all.* Higher scores indicated more self-efficacy.
 
*Willingness and Intention to Use PrEP*. This measure was assessed with two questions: “If PrEP is effective in reducing the risk of HIV by 90%, and in the next 12 months PrEP was offered for free in Colombia, would you like to use PrEP to prevent HIV?” and “If in the next 12 months, your doctor or other health professional was available for a PrEP prescription, would you start taking the PrEP pills?” The questions were rated on a 5-point scale ranging from 1 = *definitely yes* to 5 = *definitely no.* Willingness and intention to use PrEP was dichotomized into two categories 1 = to definitely yes and probably yes and 0 = all other answer options: neutral, probably not, definitely not.

Before introducing respondents to PrEP scales, a summary of the meaning of PrEP was presented as follows.

“Preexposure prophylaxis (PrEP) is an HIV infection prevention strategy where HIV-negative individuals take anti-HIV medications before coming into contact with HIV to reduce their risk of becoming infected. Medications prevent HIV from establishing an infection within the body. PrEP has been shown to reduce the risk of HIV infection through sexual contact in gay and bisexual men, transgender women, and heterosexual men and women, as well as in people who inject drugs. It does not protect against other sexually transmitted infections (STIs) nor does it prevent pregnancy. It is not a cure for HIV. Using tenofovir/emtricitabine–TDF/FTC as PrEP provides a 96% to 99% reduction in the risk of infection in HIV-negative people who take the pills every day as directed. If you miss a daily dose, the level of protection against HIV may decrease. It only works if you take the medicine. People who use PrEP correctly and consistently have higher levels of protection against HIV.”

### 2.4. Statistical Analyses

Descriptive statistics were used to summarize the social characteristics of the sample. The next step in the analysis was to perform an exploratory factor analysis with a random sample of 50% of participants. In interpreting the factor pattern, a factor loading ≥0.40 was considered good. For this analysis, a polychoric correlation matrix was used. Internal consistency and reliability were tested using Kuder-Richardson formulas and Cronbach's coefficients. Then, confirmatory factor analysis was conducted with the other half of the sample to assess the model fit of the exploratory analysis. The model fit was assessed using traditional fit indices [15]. We performed an item response theory analysis to identify the degree of difficulty of the items of the knowledge scale. The associations between variables were done using Spearman's correlation analysis and confidence interval-based estimation of relevance (CIBER) analysis [16]. All analyses were performed using Stata/IC version 16 and R version 4.0.2.

## 3. Results

The baseline demographic characteristics of the 287 participants are shown in Table 1.

### 3.1. Distribution of Items in the Scales

A total of 72% said they had heard of PrEP and indicated they had a low-moderate level of knowledge about PrEP. Participants knew the purpose of PrEP (78%), its efficacy (74%), and that it does not prevent other sexually transmitted infections (72%), but their knowledge on the importance of monitoring, adherence, and its relationship with HIV status was low. A high percentage of positive attitudes toward PrEP was observed. Overall levels of PrEP stigma were low. The descriptive norms items showed that participants think their communities and friends would like to learn about PrEP. In terms of subjective norms, the level of support to use PrEP seemed moderately high. Levels of self-efficacy varied across items, with more difficulty reported on items related to paying for PrEP and visiting a doctor (data not shown).

### 3.2. Item Response Analysis and Factor Analysis

In the item response analysis for the PrEP knowledge scale, only one item failed to demonstrate an acceptable fit (“PrEP can be taken by people who already have HIV,” *p*=0.038). Four items of the knowledge scale showed a high degree of difficulty and did not show acceptable discrimination values (Table 2). The exclusion of the poor fit item and the difficult four items represented an increase in the mean of knowledge from 52/100 points to 63/100 points (data not shown).

Exploratory factorial analysis (Table 3) revealed three factors for PrEP knowledge, a unique factor for PrEP attitudes, PrEP stigma, and PrEP subjective norms, and two factors for the PrEP descriptive norms and PrEP self-efficacy. The Cronbach's alpha coefficients for the scales were between 0.70 and 0.86. The confirmatory factor analysis of the scales indicated that all items of the attitude scale had a good fit; for the other scales, the exclusion of one or two items increased the fit of the scales (Table 4).

### 3.3. Intention to Use PrEP: CIBER Analysis by Items Scales for PrEP

Two knowledge items were related to willingness and intention: “PrEP can be taken by people who already have HIV” and “You must take an HIV test every 3 months while taking PrEP.” Additionally, two were related with intention: “The PrEP pill contains two medicines that are also used to treat HIV” and “Daily PrEP use can lower the risk of getting HIV from sex by more than 90%.” In the case of attitudes and subjective norms, all items were related to intention and willingness in the direction expected: more positive attitudes were related to greater willingness and intention. Concerning stigma, strong negative associations were found for all items, and from the descriptive norms scale, only one item was not related to both outcomes: “My friends would be interested in learning more about PrEP.” Concerning subjective norms, all items were related to willingness and intention, and for the items related to self-efficacy, two items were strongly related to willingness to use PrEP (being able to take the medicine and being able to visit a doctor for monitoring) and one item was strongly related to intention (being able to take the medicine). Interestingly, participants reporting less difficulty in paying were less likely to have intention. The *R* square was higher for the attitudes scale with 0.15 for willingness and 0.14 for intention to use PrEP (data not shown).

#### 3.3.1. Correlations

**Figure 1:**
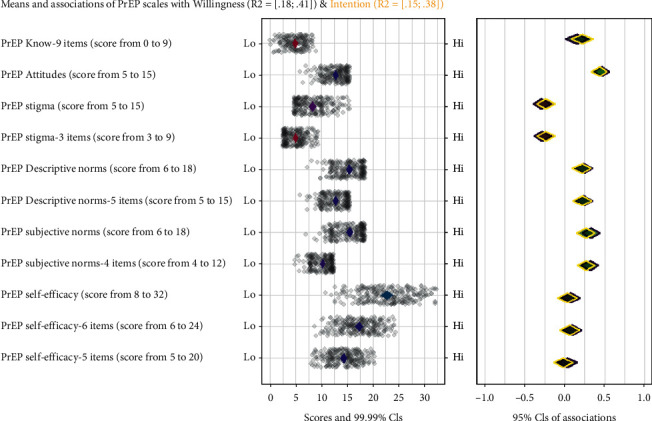
The output of the confidence interval-based estimation of relevance (CIBER) analysis regarding determinants of willingness (purple color) and intention (yellow color) to use PrEP among MSM. Diamonds in the left-hand panel indicate the means and corresponding 99% confidence interval of each scale's scores (scales of PrEP attitudes, stigma, descriptive norms and subjective norms, and PrEP self-efficacy). Green diamonds represent MSM who have a willingness and intention to use PrEP, and purple diamonds represent those who did not. Diamonds in the right-hand panel represent the 95% confidence intervals of the associations (Cohen's d) between each determinant and willingness and intention to use PrEP. Willingness and intention to use PrEP” was dichotomized into two categories: 1 = to definitely yes and probably yes and 0 = all other answer options: neutral, probably not, definitely not.

Positive and moderate correlations were found between the scales. The PrEP knowledge scale was correlated to PrEP attitudes (*r* = 0.40) and subjective norms scales (*r* = 0.27) and negatively with PrEP stigma (*r* = −0.18). The attitudes scale correlated to the stigma scale (*r* = −0.29), descriptive norms (*r* = 0.20), subjective norms (*r* = 0.45), and self-efficacy (0.15 < *r* < 0.25). The stigma scale correlated with descriptive norms (−0.17 < *r* < −0.20), subjective norms (−0.47 < *r* < −0.52), and self-efficacy (−0.18 < *r* < −0.37). Descriptive norms correlated to subjective norms positively (0.32 < *r* < 0.37). Finally, subjective norms were correlated positively with the self-efficacy shorter scale (*r* = 0.18). Willingness and intention to use PrEP correlated positively with PrEP knowledge (*r* = 0.23), attitudes (0.40 < *r* < −0.42), descriptive norms (0.25 < *r* < 0.31), and subjective norms (0.24 < *r* < 0.33) and inversely with stigma scales (−0.24 < *r* < −0.32). No correlation was found between self-efficacy scale and willingness or self-efficacy and intention (Figure 1).

## 4. Discussion

In our MSM study in Colombia, results indicated that the majority of the items and the scales developed by Walsh [14] are reliable and show a good fit. Although a reduction of items seems to favour a better fit in most of the scales, the majority of the relationships among constructs of the IBM model were supported by the data: better-informed participants have positive attitudes, more motivated (less stigma with more positive norms) participants have higher levels of self-efficacy, and those with more positive attitudes and less stigma had more intention to use PrEP. Future use must consider the level of difficulty of the items of the knowledge scale, and the self-efficacy scale needs to be refined and consider additional self-efficacy items.

The knowledge scale presented a high degree of difficulty for participants, especially items describing the relationship between HIV and the need for extra monitoring. Interestingly, the items with a greater level of difficulty were correlated with willingness and intention. This particular finding favours the need to provide accurate and relevant knowledge of PrEP [17, 18]. Consistent with other studies, knowledge of PrEP was related to intention to use PrEP [6], and knowledge was related to higher positive attitudes and less stigma [19, 20]. Thus, health education within MSM communities on PrEP may need to emphasize the use of PrEP exclusively in HIV-negative individuals, the importance of monitoring and follow-up, and the potential side effects associated with PrEP.

Self-efficacy is a crucial element in developing strategies for prevention in HIV [8], including PrEP uptake; however, we did not find a correlation of the scale with intention and willingness to use PrEP. Although Walsh's validation study and others have found self-efficacy as an important construct for PrEP uptake, in other populations, self-efficacy has not been associated with HIV risk behaviours [21]. In our sample, only two items were related to intention and willingness to use PrEP: the degree of difficulty in taking the medication and attending the monitoring appointment. This finding conveyed the significance of adherence to treatment and access to healthcare as facilitators of PrEP use [22, 23]. This is not a surprising result, as access to treatments, care, and HIV prevention in Colombia is fragmented, limited, and problematic due to the organization of its health system [24, 25]. One possible explanation for the lack of association of the self-efficacy scale and the outcomes is the choice of the items of the scale. The difficulty of assessing self-efficacy in the context of complex behaviours has been recognized [26]. PrEP uptake skills include consulting a provider, discussing PrEP, discussing sexual health, and adhering to condom use, medication, and monitoring and HIV testing. The self-efficacy scale comprised items related to discussions with providers about sexual health or having access to HIV testing, which participants may not relate directly to taking pills.

Interestingly, having difficulty paying for the medication was related to greater intention, a finding that contrasts with other studies that found the cost of PrEP was a detrimental barrier to PrEP uptake [18, 27]. A study in Brazil found that 75.8% of the participants reported they would use PrEP even if they had to pay for it [28]. This result could be explained by participants' positive attitudes toward PrEP and its effectiveness in reducing HIV infection.

In support of the IMB model, our data indicated that the motivation construct had good construct validity and is relevant for PrEP intention in the sample. The attitudes toward PrEP were the strongest predictor of willingness and intention to use PrEP. The attitudes scale presented a good fit and included attitudes toward adherence, effectiveness, and safety, which are important aspects related to PrEP uptake [14]. The attitudes scale also showed a high correlation with knowledge and a moderate correlation with self-efficacy, supporting the IMB model results again [8].

As a motivation construct in the IMB model, PrEP stigma is a clear barrier for PrEP uptake in our study as it has been observed in other settings [14, 29]. Yet, overall levels of stigma in our sample were not high, with participants more concerned about family members' attitudes toward taking PrEP than friends' attitudes. These results could be explained by MSM's greater knowledge and exposure to information on HIV [30]. This was expected to happen in a sample that had access to the internet and was relatively well informed [31].

The descriptive and subjective norms had a good fit and worked well in our study population. PrEP descriptive and subjective norms were positively associated with willingness and intention to uptake PrEP and knowledge, attitudes, and self-efficacy. This result means that MSM would be more motivated to use PrEP if friends and sexual partners had positive attitudes and views about PrEP. The results also highlighted an important correlation between the stigma scale and the scales related to norms, which agrees with results in other contexts [32].

These validated scales in Spanish language may prove useful in both the clinical and public health domains. In the clinic, the assessment of potential PrEP users involves decision-making by the patient about taking or declining PrEP. Here, scientific evidence needs to be presented to the patient to inform their decision. In this instance, the knowledge scale items could be introduced at different stages of the interaction patient-healthcare provider to ensure key items of PrEP knowledge are delivered to and assimilated by PrEP users. Similarly, the attitude scale can help determine if the education delivered in the clinic has impacted PrEP candidates' attitudes toward PrEP. In addition, some items of the stigma scale can help recognize situations where healthcare providers need to intervene to reinforce positive messaging regarding PrEP use. In the public health domain, agencies can use these scales to monitor the trends of PrEP knowledge, attitudes, stigma, norms, and self-efficacy over time in populations of interest following PrEP implementation and detect areas where improvement or adjustments are needed or whether PrEP campaigns are reaching goals.

### 4.1. Limitations and Strengths

Our validation sample was composed of MSM living in 25 cities in Colombia recruited through different media and using an online survey. This sample may not reflect the sociodemographic or sexual practices of all MSM in Colombia. The sample of participants was highly educated and with favourable socioeconomic statuses. They were also recruited via social media, which may have excluded those that do not use these resources or have access to them. Despite this, our results were consistent with the IMB model, and the scales show good reliability and construct validity. We observed high attitudes, low stigma levels, and high self-efficacy. Although our validation results suggested that the translation of scales was adequate for this sample, further studies may need to test our Spanish version or adapt the Spanish language to other settings given variations in the expressions in different countries and regions. Our previous work on translating HIV stigma scales has demonstrated the usefulness of having different Spanish translations [33].

## 5. Conclusions

This is the first study in Colombian MSM assessing constructs that could be incorporated into future PrEP interventions in MSM. The results indicate the importance of addressing knowledge, stigma, social norms, and positive attitudes toward PrEP and reducing barriers to the healthcare system. Results offer insights into the relationships between motivations and PrEP intentions and can provide a foundation for the development of interventions for PrEP uptake. The present results suggest that providing accurate information will be essential in communicating about PrEP in future education campaigns. The applicability of the IMB model to PrEP in this study needs to be examined in other populations and include contextual and syndemic factors known to be related to HIV risk behaviours in MSM populations [34]. The PrEP scales, especially those related to knowledge, self-efficacy, and attitudes could be used to tailor education initiatives in clinical settings providing PrEP [35]. Some recent examples suggest that some of the PrEP scales could be converted into assessment tools in the clinic to assist PrEP-related care provision or used by public health authorities to monitor trends over time [36].

## Figures and Tables

**Table 1:** Baseline demographic characteristics of the study participants.

Age, years (means; sd)	31.2; 8.9
	*n* (%)
Biological sex (men)	287 (100)
*Gender*	
Male	279 (97.2)
Nonconforming gender	3 (1.0)
Other	3 (1.0)
Prefer not to answer	2 (0.7)

*Sexual orientation*	
Heterosexual	3 (1.0)
Gay men	238 (82.9)
Bisexual	45 (15.7)
Prefer not to answer	1 (0.3)

*Civil status*	
Married—common law	45 (15.7)
Single	236 (82.2)
Separated-widow	6 (2.1)

*Education level*	
Primary school	1 (0.3)
Secondary school	26 (9.1)
Technical-superior	260 (90.6)

*Socioeconomic stratum*	
One-two (very low-low)	83 (28.9)
Three-four (middle)	166 (57.8)
Five-six (high)	39 (13.6)

*Occupation status (one or more options)*	
Work	184 (64.1)
Housekeepers	9 (3.1)
Voluntary	11 (3.8)
Student	59 (20.6)
Unemployed	62 (21.6)
Other	16 (5.6)

*Current monthly income*	
No income	44 (15.3)
<1	59 (20.6)
Between 1 and 2	67 (23.3)
>2	117 (40.8)
Health insurance/coverage (yes)	246 (86.0)
Willingness to use PrEP (definitely yes)^*∗*^	187 (71.1)
Intention to start PrEP (definitely yes)^*∗*^	167 (63.7)

^*∗*^Willingness and intention to use PrEP was dichotomized into two categories: 1 = to definitely yes and probably yes and 0 = all other answer options: neutral, probably not, definitely not.

**Table 2:** Item response analysis—PrEP knowledge scale.

Item	Difficulty parameters	Chi sq	df	*p* value	Outfit MSQ	Infit MSQ	Outfit t	Infit t	Discrim
PrEP is a daily pill you can take to reduce your risk of becoming infected with HIV^*∗*^	0	168.769	200	0.947	0.840	0.899	−0.923	−1.056	0.35
You should not use PrEP if you don't know your HIV status +	1.969	170.56	200	0.935	0.849	0.925	−1.503	−1.178	0.397
If you do not take PrEP consistently, there may not be enough medicine in your bloodstream to block the HIV virus	0.965	207.431	200	0.344	1.032	0.984	0.348	−0.226	0.327
PrEP can be used to prevent STIs like gonorrhea, chlamydia, syphilis, herpes, and HPV	0.329	191.344	200	0.658	0.952	0.952	−0.296	−0.560	0.327
If you start taking PrEP, you will have to take it for the rest of your life	0.965	224.213	200	0.115	1.098	1.098	1.141	1.455	0.163
PrEP can be taken by people who already have HIV^*∗∗*^	2.208	236.976	200	0.038	1.043	1.043	1.477	0.646	0.169
You must take an HIV test every 3 months while taking PrEP +	1.831	193.298	200	0.620	0.994	0.994	−0.364	−0.079	0.301
There are many serious side effects of taking PrEP +	2.622	196.265	200	0.561	1.030	1.030	−0.108	0.398	0.206
The PrEP pill contains two medicines that are also used to treat HIV +	2.135	167.826	200	0.953	0.867	0.867	−1.510	−2.063	0.446
Daily PrEP use can lower the risk of getting HIV from sex by more than 90%	0.244	146.976	200	0.998	0.870	0.870	−1.936	−1.553	0.437

^*∗*^The difficulty parameter of this item had been fixed to 0. ^*∗∗*^Items of the scale had a bad fit. +Items of the scale show a high degree of difficulty and did not show acceptable discrimination.

**Table 3:** Factor loadings for item scale versions (pattern matrix).

Scale items	PrEP known factor solution, 9 items			PrEP attitudes factor solution	PrEP stigma factor solution	PrEP descriptive norms factor solution^*∗*^		PrEP subjective norms factor solution	PrEP self-efficacy factor solution	
	(F I)	(F II)	(F III)	(F I)	(F I)	(F I)	(F II)	(F I)	(F I)	(F II)
PrEP is a daily pill you can take to reduce your risk of becoming infected with HIV	**0.60**									
You should not use PrEP if you don't know your HIV status	**0.60**									
If you do not take PrEP consistently, there may not be enough medicine in your bloodstream to block the HIV virus			0.66							
PrEP can be used to prevent STIs like gonorrhea, chlamydia, syphilis, herpes, and HPV		**0.66**								
If you start taking PrEP, you will have to take it for the rest of your life			0.24							
PrEP can be taken by people who already have HIV	**0.37**									
You must take an HIV test every 3 months while taking PrEP		**0.36**								
There are many serious side effects of taking PrEP			0.49							
The PrEP pill contains two medicines that are also used to treat HIV		**0.41**								
PrEP is effective at preventing HIV				**0.76**						
People who take PrEP are responsible				**0.75**						
Taking PrEP is safe				**0.85**						
It would be no trouble to take PrEP every day				**0.63**						
The government makes certain that drugs like PrEP are safe				**0.68**						
Getting a PrEP prescription from a doctor would be embarrassing^*∗*^					**0.77**					
People who take PrEP are promiscuous^*∗*^					**0.73**					
I would be concerned if my family found out I was taking it					**0.86**					
I would be concerned if my friends found out I was taking it					**0.91**					
I would be concerned if my sexual partner(s) found out I was taking it					**0.86**					
People in my community would be interested in learning more about PrEP^*∗*^							**0.85**			
People in my community would be willing to talk with their doctors about using PrEP							**0.95**			
People in my community would consider taking PrEP							**0.92**			
My friends would be interested in learning more about PrEP						**1.00**				
My friends would be willing to talk with their doctors about using PrEP						**0.94**				
My friends would consider taking PrEP						**0.86**				
My friends would be supportive of me using PrEP								**0.70**		
My friends would think it was smart if I used PrEP^*∗*^								**0.84**		
My friends would think it was responsible if I used PrEP^*∗*^								**0.90**		
My sexual partner(s) would be supportive of me using PrEP								**0.87**		
My sexual partner(s) would think it was smart if I used PrEP								**0.85**		
My sexual partner(s) would think it was responsible if I used PrEP								**0.85**		
How difficult would it be for you to seek out more information about PrEP to decide if it is right for you?									**0.67**	
How difficult would it be for you to talk with your sexual partner(s) about the decision to take PrEP?									**0.64**	
How difficult would it be for you to visit a doctor who can provide PrEP?^*∗*^									**0.82**	
How difficult would it be for you to talk openly and honestly with a doctor about your sexual behaviours?^*∗*^									**0.78**	
How difficult would it be for you to get tested for HIV?									**0.56**	
How difficult would it be for you to find a way to pay for PrEP?									**0.58**	
How difficult would it be for you to take medicine like PrEP every day?^*∗*^										**0.93**
How difficult would it be for you to visit a doctor every three months for routine screenings?										**0.87**
**Proportion variance**		0.33		0.54	**0.69**	**0.63**	**0.23**	**0.70**	**0.39**	**0.18**

^*∗*^Scale items had a bad fit and were excluded. Knowledge PrEP scale: each item was scored on a 3-point scale: 1 = true, 2 = false, and 3 = don't know. Scores on this scale were recoded as 1 = correct and 0 = incorrect/don't know, with higher scores indicating a greater degree of knowledge. Each item was scored on a 3-point scale as 1 = disagree, 2 = neutral, and 3 = agree, to scales of attitudes, stigma, descriptive norms, and subjective norms, with higher scores indicating more positive attitudes, a greater degree of stigma, and more positive norms. And for the self-efficacy scale, each item was scored on a 4-point scale ranging from 1 = very difficult to 4 = not difficult at all. Higher scores indicated more self-efficacy.

**Table 4:** The confirmatory factor analysis of the scales (CFA).

Model-PrEP scale	Items		CFA fit, confirmatory sample (*n* = 240)							
	Initial	Final	KMO	BTS (*p* value)	CFI	TLI	SRMR	RMSEA	Prob > chi2-excluding item	Cronbach's alpha coefficients
PrEP attitudes	5	5	0.749	<0.001	1.00	1.01	0.03	0.00	LR test of model vs. saturated: chi2 (5) = 4.71, prob > chi2 = 0.4517	0.70
PrEP stigma	5	5	0.7814	<0.001	0.90	0.79	0.06	0.18	LR test of model vs. saturated: chi2 (5) = 26.18, prob > chi2 = 0.0001	0.81
PrEP stigma (excluding two items)	5	3	0.68	<0.001	1.00	1.00	0.00	0.00	LR test of model vs. saturated: chi2 (0) = 0.00, prob > chi2 =	0.81
PrEP descriptive norms^*∗*^	6	6	0.76	<0.001	0.94	0.90	0.05	0.15	LR test of model vs. saturated: chi2 (8) = 31.79, prob > chi2 = 0.0001	All: 0.8386 subitems: 0.8094; 0.8597
PrEP descriptive norms (excluding one item)	6	5	0.79	<0.001	1.00	1.02	0.01	0.00	R test of model vs. saturated: chi2 (4) = 1.56, prob > chi2 = 0.8158	All: 0.8259 subitems: 0.8094; 0.8597
PrEP subjective norms	6	6	0.78	<0.001	0.900	0.834	0.059	0.180	LR test of model vs. saturated: chi2 (9) = 47.19, prob > chi2 = 0.0000	0.864
PrEP subjective norms (excluding two items)	6	4	0.69	<0.001	0.997	0.990	0.020	0.049	LR test of model vs. saturated: chi2 (2) = 2.63, prob > chi2 = 0.2681	0.810
PrEP self-efficacy^*∗*^	8	8	0.65	<0.001	0.793	0.694	0.102	0.153	LR test of model vs. saturated: chi2 (19) = 78.46, prob > chi2 = 0.0000	All: 0.7423; subitems: 0.7290; 0.7601.
PrEP self-efficacy^*∗*^ (excluding two items)	8	6	0.57	<0.001	0.948	0.903	0.069	0.078	LR test of model vs. saturated: chi2 (8) = 14.53, prob > chi2 = 0.0689	All: 0.6283 subitems: 0.5379; 0.7601
PrEP self-efficacy^*∗*^ (excluding three items)	8	5	0.61	<0.001	1.000	1.091	0.033	0.000	LR test of model vs. saturated: chi2 (4) = 2.03, prob > chi2 = 0.7298	All: 0.5628; subitems: 0.5379;

^*∗*^Two factors were obtained. A good fit is indicated by CFI and TLI values greater than 0.95 and RMSEA values less than 0.05 and acceptable fit by CFI and TLI values over 0.90 and RMSEA values less than 0.06. ^#^Chi^2^ cannot be estimated because the model fits three items.

## Data Availability

The data tables used to support the findings of this study are available upon request to the corresponding author.

## Supplementary Materials

Supplementary Materials Supplementary Table 1: knowledge PrEP scale—imputation of nonassigned items.
