# Supplementary material for: Scales to Assess Knowledge, Motivation, and Self-Efficacy for HIV PrEP in Colombian MSM: PrEP-COL Study
Source: AIDS Res Treat. 2021 Sep 7;2021:4789971. doi: 10.1155/2021/4789971 (PMC8443363; doi:10.1155/2021/4789971)
Supplement: Supplementary Materials — Supplementary Table 1: knowledge PrEP scale—imputation of nonassigned items. [file 4789971.f1.docx]

Supplementary file-table 1. Knowledge PrEP scale - imputation of non-assigned items

|  | **Total - n=112-130**  **(**non-assigned items) | | **Total - n=202-**  (imputation*) | | **p-value** | |
| --- | --- | --- | --- | --- | --- | --- |
| **Items** | **(%)** | **(%)** | |  | |  |
| PrEP is a daily pill you can take to reduce your risk of becoming infected with HIV. (T) | 77.9% | 78.2% | | 0.9439 | |  |
| You should not use PrEP if you don’t know your HIV status. (T) | 39.5% | 38.6% | | 0.8712 | |  |
| If you do not take PrEP consistently, there may not be enough medicine in your bloodstream to block the HIV virus. (T) | 58.1% | 60.4% | | 0.6772 | |  |
| PrEP can be used to prevent STIs like gonorrhea, chlamydia, syphilis, herpes, and HPV. (F) | 76.1% | 72.8% | | 0.5179 | |  |
| If you start taking PrEP, you will have to take it for the rest of your life. (F) | 60.0% | 60.4% | | 0.944 | |  |
| PrEP can be taken by people who already have HIV (F) | 35.7% | 33.7% | | 0.714 | |  |
| You must take an HIV test every 3 months while taking PrEP. (T) | 41.0% | 41.6% | | 0.9222 | |  |
| There are many serious side effects of taking PrEP. (F) | 25.6% | 25.7% | | 0.9739 | |  |
| The PrEP pill contains two medicines that are also used to treat HIV. (T) | 35.1% | 35.2% | | 0.9913 | |  |
| Daily PrEP use can lower the risk of getting HIV from sex by more than 90%. (T) | 70.9% | 74.3% | | 0.5002 | |  |

T: True; F: False. To reduce the burden of the number of questions, participants were randomly assigned to 70% of the items of the knowledge scale. *Multiple imputation was developed.
